# Pentraxin 3: a powerful orchestrator in urinary tract infection

**DOI:** 10.7150/ntno.60901

**Published:** 2021-05-12

**Authors:** Cheng Qiu, Tianyi Liu, Dan Luo, Zhaoxiang Xie

**Affiliations:** 1 Department of Orthopaedic Surgery, Qilu Hospital, Cheeloo College of Medicine, Shandong University, Jinan 250012, Shandong, P. R. China.; 2 Cheeloo College of Medicine, Shandong University, Jinan 250012, Shandong, P. R. China.; 3 College of Stomatology, Qingdao University, Qingdao 266071, Shandong, P. R. China.; 4 Department of Urology, Sun Yat-Sen Memorial Hospital, Sun Yat-Sen Memorial Hospital, Sun Yat-Sen University, Guangzhou 510275, Guangdong, P. R. China.

Urinary tract infection (UTI) is a common disease in clinic. According to early reports, women are the major parts and usually long-term afflicted with UTI 1. Infections of the urogenital tract are mainly attributed to the migration and colonization of pathogens from rectum and perineum 2. The microorganisms, causing UTI, consist of Escherichia coli (E.coli), Klebsiella, Enterococcus, Pseudomonas or others. However, mounting evidence demonstrated that E.coli is the main strain to induce UTI 3. Increasing age and frequent intercourse as well as diabetes are stimulating factors for UTI, so are obesity and female anatomy 2. The typical clinical symptoms of UTI including hematuria, dysuria, frequent urination, urgent urinatione, etc.

PTX3 is a multifunctional protein polymers as the first member of the long pentraxin family 4, 5. The sequence, regulating function as well as ligand discernment of PTX3 are greatly conservative during human evolution 6. It is shown that PTX3 binds to selected pathogens, induce complement activation and regulate inflammatory responses. Hence, we summarized the mechanism of PTX3 in UTI (**Figure 1**). Herein, we speculate the process of PTX3 binding E.coli given the potential to affect inflammatory reaction by the aid of neutrophils and perhaps additional immune cells 7. During UTI, E.coli, inflammatory cytokines (i.e., IL-1β and TNF-α) and Toll-like receptor agonists or microbial components could induce several cells like as urothelial cells and kidney cells to secrete PTX3 3, 8. Meanwhile, leukocytes and urothelial cells are identified to the main provenance to produce and release PTX3 locally in the urinary tract 3. In the early phase of inflammatory reaction, neutrophils are the earliest to be recruited and then infiltrate into inflammatory environment 9. As a repository of PTX3, neutrophils promptly release PTX3 which temporally previous to other cells producing PTX3 by gene expression. Parts of released PTX3 enter neutrophil extracellular structure, an extracellular DNA fibrillary network (NETs) formed by neutrophils squeezing out nuclear content to form, trapping microorganisms and reserving antimicrobial components. In urinary systematic infection, E.coli attaches to the superficial epithelium of the urinary tract *via* TLR4 molecules locating in the epithelium 10. Following activation of TLR4 is prone to promote the MyD88 signaling cascade burst that triggers the gression of NF-κB transcription factor into the nucleus at the downstream. Thereafter, PTX3 is secreted into the urinary tract to bond bacteria so that facilitate phagocytosis, microbial recognition and phagosome maturation of the neutrophils as well as activate the classic pathway of complement 6.

In innate immunity, PTX3 activates the classical complement pathway *via* combining C1q with the globular head, the first member of the cascade 8. Only on the condition of mimics C1q binding to a microbic surface, C1q being plastic-immobilized, PTX3 could combine with C1q to trigger the classical complement pathway 11. Whereas in the senario of fluid phase, there is a possible dose-dependent prohibitive effect of C1q hemolytic activity by competitive obstructing of related sites (**Figure 2**) 3, 8, 9. The third exon-encoded pentraxin domain of PTX3 is of capacity to bind with P-selectin on its N-linked glycosidic moiety 6. Hence, PTX3, as a negative regulating mediator, restraining undue P-selectin-dependent assemblage of neutrophil granulocytes. When massive leukocyte activated, PTX3 could be released to function locally as a negative feedback agent to lessen neutrophil recruitment. Under different scenarios of localized inflammation and systemic inflammatory reactions, PTX3 plays asymmetric roles involving in inflammatory reactions 12. In the former reactions, neutrophils degranulate to augment local PTX3 in the microcirculation to decrease excessive recruitment *via* binding P-selectin with a low dissociation rate. On the contrary, degranulation of neutrophils and gene expression result in high level of circulating PTX3 to serve as a negative feedback loop systemically under the latter scenario.

Taken together, PTX3 plays a critical role in these parts comprising pathogens recognition, immune cells recruitment, inflammation burst and pathogens clearance. However, the underlying mechanisms of UTI are complicated and still poorly understood that need to be elucidated. PTX3 seems as a powerful orchestrator during this process and interacts with a huge molecule network that functions in the UTI. A recent study showed that urinary PTX3 also increased in patients with scarred kidneys and speculated that might be helpful to predict renal parenchymal scar due to past pyelonephritis 13. Therefore, we hereof propose that the expression of PTX3 might be regarded as a prognosis biomarker for UTI. Lower level of PTX3 may represent infection-free after treatment. Further clinical research need to focus on the molecular detection of PTX3 on patients of UTI both locally and systematically.

## Figures and Tables

**Figure 1:**
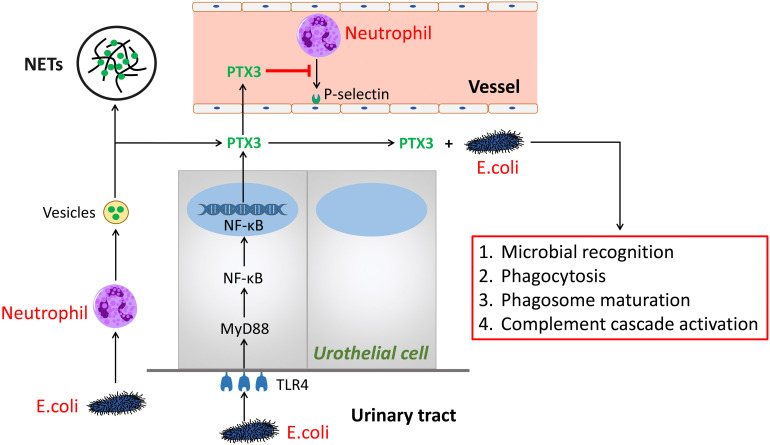
** The role of PTX3 in urinary tract infection.** In the early phase of urinary tract infection, E.coli stimulates neutrophils releasing vesicles which enter the extracellular environment and release of PTX3. One part of PTX3 participate in constructing extracellular DNA fibrillary networks. Additional part of PTX3 inhibit neutrophils binding P-selectin and combine with E.coli to promote the processes of microbial recognition, phagocytosis, phagosome maturation, as well as complement cascade activation, so do PTX3 produced by the urothelial cells via the TLR4/MyD88/NF-κB signaling pathway.

**Figure 2:**
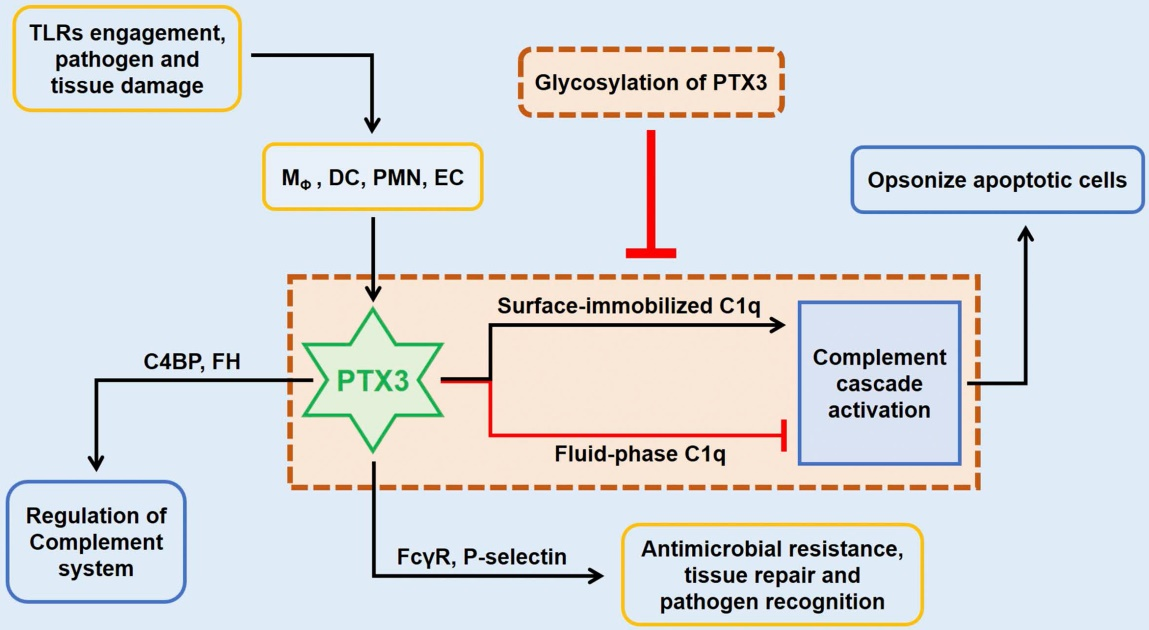
** The role of PTX3 in innate immunity.** In innate immunity, the upstream lineage producing PTX3 relies on the activation of pattern recognition molecules (PMNs), dendritic cell (DC), neighboring macrophages and endothelial cells (ECs) when stimulated with TLRs engagement or pathogen recognition or tissue damage. By targeting on FcγR and P-selectin, the tremendously increased PTX3 participates in antimicrobial resistance and tissue repair, and also facilitates pathogen recognition and removal. The preliminary role of PTX3 is to function the intermediary role in complement cascade by interacting with factor H (FH) and C4 binding protein (C4BP). In addition, the culmination occurs when the interaction between PTX3 and surface immobilized C1q results in the activation of the classical complement cascade which aims to opsonize apoptotic cells. However, fluid-phase binding of PTX3 to C1q inhibits the activation of complement system by blocking relevant sites, whilst this pipeline is suppressed by the glycosylation of PTX3.
